# Distribution and Potential Ecological Risk of Heavy Metals in Water, Sediments, and Aquatic Macrophytes: A Case Study of the Junction of Four Rivers in Linyi City, China

**DOI:** 10.3390/ijerph16162861

**Published:** 2019-08-10

**Authors:** Xiuling Li, Henglun Shen, Yongjun Zhao, Weixing Cao, Changwei Hu, Chen Sun

**Affiliations:** 1 College of Life Sciences, Linyi University, Linyi 276000, China; 2 College of Life Sciences, Zaozhuang University, Zaozhuang 277160, China; 3 College of Biological Chemical Science and Engineering, Jiaxing University, Jiaxing 314001, China

**Keywords:** heavy metal, sediment, river, risk assessment, bioaccumulation, bioindicator

## Abstract

The Yi River, the second longest river in Shandong Province, China, flows through Linyi City and is fed by three tributary rivers, Beng River, Liuqing River, and Su River in the northeastern part of the city. In this study, we determined the concentrations of five heavy metals (Cr, Ni, Cu, Zn, and Pb) in water, sediment, and aquatic macrophyte samples collected from the junction of the four rivers and evaluated the potential ecological risk of heavy metal pollution. Most of the heavy metals in water were in low concentrations with the water quality index (WQI) below 1, suggesting low metal pollution. The sediments showed low heavy metal concentrations, suggesting a low ecological risk based on the potential ecological risk index (RI) and the geo-accumulation index (*I_geo_*). The aquatic plant species *Potamogeton crispus* accumulated considerable amounts of heavy metals, which were closely related to the metal concentrations of the sediment. The plant species *Salvinia natans* also showed an excellent metal accumulation capability. Based on our results, the junction of the four rivers is only slightly polluted in terms of heavy metals, and the plant species *P. crispus* is a suitable bioindicator for sediment heavy metal pollution.

## 1. Introduction

Rivers, especially those flowing through urban areas, play a key role in preserving freshwater, adjusting the local climate, and improving the environmental conditions [1]. However, with accelerated population growth, urbanization, and industrialization, heavy metal pollution of rivers has become a serious issue. In 2007, approximately 90% of the urban rivers in China were polluted, with 900 tons of heavy metals being transported into freshwater bodies [2]. Anthropogenic activities, such as fertilization, inadequate industrial effluent disposal, oil spillage, domestic sewerage disposal, and mineral mining, as well as rain water and atmospheric deposition, are particularly responsible for the increasing accumulation of heavy metals in water, sediments, and macrophytes, significantly contributing to the pollution of aquatic ecosystems [3,4]. The pollution of water bodies with heavy metals seriously threatens aquatic biodiversity because of the toxicity, persistence, bioaccumulation, and non-degradability of heavy metals, and contaminated drinking water represents a severe health hazard in humans [5].

Aquatic macrophytes can accumulate and concentrate large amounts of various substances and can act as bio-filters by accumulating heavy metals from the surrounding environment, making them excellent indicators of heavy metal contamination in aquatic ecosystems [6,7]. Peng et al. found that the aquatic plants *Potamogeton pectinatus* had high accumulation capacity for cadmium (Cd), lead (Pb), copper (Cu), zinc (Zn), and manganese (Mn) with 596, 318, 62.4, 6590, and 16,000 mg/kg (DW), respectively [8]. *Salvinia minima* was shown to accumulate metals within tissues when cultivated in higher concentrations of selected heavy metals, with maximum removal rates of 0.0045, 0.0595, 0.1423, and 0.4046 mg/m^2^·day for Cd, nickel (Ni), Pb, and Zn, respectively [9]. The analysis of water, sediment, and macrophyte samples can be performed to assess the overall heavy metal pollution and the impact of heavy metals on aquatic ecosystems. As carriers of contaminants, sediments are generally recognized as a primary sink for heavy metals in aquatic environments; more than 85% of heavy metals eventually deposit on surface sediments [10].

The investigation of heavy metal concentrations and distribution is useful to determine the pollution level in aquatic environments and to provide basic information for the assessment of environmental health risks [11]. The most commonly cited assessment indices in environmental studies include the water quality index (WQI), the potential ecological risk index (RI), the index of geo-accumulation (*I_geo_*), and the bioconcentration factor (BCF), which are widely used to evaluate heavy metal pollution in water, sediments, and macrophytes. To more deeply understand the issue of heavy metal pollution of water bodies, the integrated application of multi-assessment methods to evaluate the ecological risk is important [12,13,14].

The Yi River (34°23′–36°20′ N, 117°25′–118°42′ E) originates from the southern foothills of the Mountain Lu in Yiyuan County, Shandong Province, and flows into the Yellow Sea from Jiangsu Province; it has a total length of 574 km and a basin area of 17,325 km^2^ in Shandong Province. As an important tributary of the Huai River, the Yi River is the second longest river in Shandong Province and the largest river in the Yimeng mountainous area. Three tributary rivers including the Beng River, the Liuqing River, and the Su River, feed into the Yi River in the northeastern part of the Linyi City, forming a junction. A rubber dam, the Xiaobudong Rubber Dam with a length of 1135 m, was built 4.5 km downstream of the junction for water storage in 1997, forming the Yimeng Lake with a water surface of 10 km^2^. The Yi River and the tributaries play important roles in flood control, irrigation, and maintenance of the ecosystems. With the growth of the population and the rapid development of the industry and agriculture, the water area in the junction of the four rivers was contaminated with heavy metals. However, the pollution levels of the water, the sediment, and aquatic organisms are still unknown, and respective studies are scarce. In recent years, river management has ever been strengthened by the local government. Most chemical, electroplating, and manufacturing factories were shut down or moved out of the river basin, but their contribution to heavy metal pollution in the past still lasted. The effluent of sewage treatment plants near the upper stream, non-point source pollution, surface runoff, and automobile exhaust are all potential sources of heavy metals in the water and sediments of the rivers nowadays.

In this context, we collected water and sediment samples as well as samples from two widely distributed aquatic plants, *Potamogeton crispus* Linn. and *Salvinia natans* L., from the junction of the four rivers in Linyi City, with the objectives to (1) investigate the contamination levels, distribution patterns, and the potential ecological risks of heavy metals in water and sediments, and (2) evaluate the effects of the inflow of the tributaries on the heavy metal level of the main stream, and (3) assess the bioaccumulation of heavy metals in aquatic macrophytes and its correlation with sediments or water.

## 2. Materials and Methods

### 2.1. Sampling Sites and Methods

**Figure 1:**
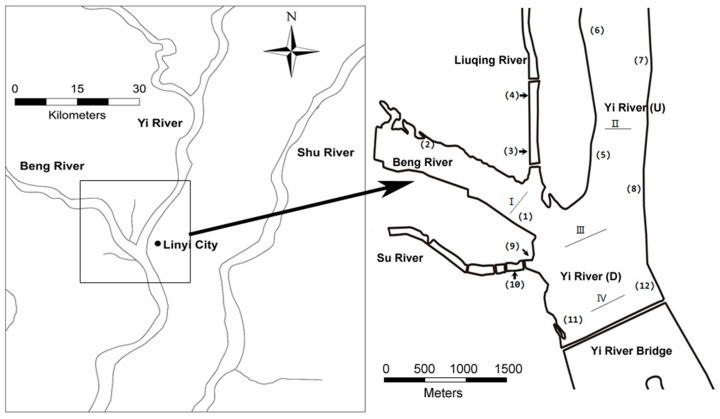
Location of sampling sites in the junction of rivers in Linyi City, China. Number (1) to (12) represent sampling sites of water and aquatic plants. Number I to IV represent sampling sites of the sediments.

Water samples were collected from 12 different areas (three sites at a distance of 50 m for each area) along the junction of Beng River, Liuqing River, Su River, and Yi River in April and September 2017 (Figure 1). The two sampling times were selected because April and September are the ends of the dry season and flood season of the Yi River, respectively. All samples were obtained at a depth of 0.5 m below the water surface, using clean polyethylene bottles (washed with hydrochloric acid and rinsed with distilled water); at each site, three water samples were collected. The samples were filtered through 0.45-μm Millipore filters and acidified with guaranteed grade nitric acid. Areas 1 and 2 represent the Beng River, areas 3 and 4 represent the Liuqing River, areas 5, 6, 7, and 8 represent the upstream of the Yi River (U), areas 9 and 10 represent the Su River, and areas 11 and 12 represent the downstream of the Yi River (D). Yi River (U) and Yi River (D) were selected as reference sites to evaluate the influence of other three rivers on the heavy metal level of the main stream.

The sediment samples were collected along four sections (Figure 1) in April 2017. Each section included three sampling sites, and three replicate samples collected at each site. In each sampling site, approximately the top 10-cm layer of the sediment was sampled using a sediment sampler (PBS-411, Wuhan, China). All samples were sealed in clean polyethylene bags, placed in a cooled box, transported to the laboratory, and air-dried. The samples were decomposed with a microwave (MWD-630, METASH, Shanghai, China) for metal determination. The sampling sites were located using a global positioning system (GPS), and the coordinates of the sites used for water and sediment sampling are provided in Tables S1 and S2, respectively.

Samples of the aquatic plant species *P. crispus* were collected from eight areas (areas 1, 2, 5, 6, 7, 8, 11, and 12) in April 2017. In the same areas, samples of the plant species *S. natans* were collected in September 2017. For each sampling site of specific area, plants with approximately 500 g fresh weight from at least three different strains were collected. All plants were rinsed with distilled water immediately after collection. In addition, sediment samples were also collected from the same eight sites containing *P. crispus*.

### 2.2. Sample Analysis

Five commonly measured heavy metals (Chromium (Cr), Ni, Cu, Zn, and Pb) were detected in all water and sediment samples. The sediment samples were digested with HF-HClO_4_, and the concentrations of the heavy metals were determined by inductively coupled mass spectrometry (ICP-MS, iCAP Q, Thermo, Waltham, MA, USA). The recovery percentage of the external standard ranged between 80 and 120% for all elements. The limits of detection were as follows: 0.02 μg/L for Cr, 0.03 μg/L for Ni, 0.01 μg/L for Cu, 0.02 μg/L for Zn, and 0.01 μg/L for Pb. Reference materials of heavy metals in simulated water standard solution (Cr: GBW(E)080403; Ni: GBW(E)080405; Cu: GBW(E)080396; Zn: GBW(E)080400; Pb: GBW(E)080399) were used to ensure data validity and the accuracy and precision of the analysis methods.

Plant tissue digestion was carried out using a method described in [15]. Briefly, the plants were oven-dried at 80 °C for 24 h to constant weight and then microwave-digested in two steps. The first step occurred in 10 mL of 16 mM HNO_3_ at 70 °C for 5 min, followed by 130 °C for another 5 min and by 150 °C for 4 min. The second step consisted of the addition of 1 mL of H_2_O_2_ at 85 °C for 5 min, followed by 130 °C for 4 min. After cooling, the samples were diluted with 1% (*v*/*v*) HNO_3_ to a final volume of 50 mL. Subsequently, the samples were filtered through 0.45-μm cellulose nitrate ultrafiltration membrane filters (Whatman, Maidstone, UK) and acidified with HNO_3_ to a pH of approximately 2.0, followed by heavy metal detection via ICP-MS.

### 2.3. Water Quality Index and Potential Ecological Risk Index of Sediments

The WQI represents the total quality of the water with respect to heavy metals [16]:

(1) $$\mathrm{WQI}=\frac{1}{n}\overset{n}{\underset{i=1}{\sum }}\frac{C_{D}^{i}}{C_{R}^{i}}$$

where *C_D_* is the measured concentration of the sample and *C_R_* is the reference value according to Class I of the environmental quality standard for surface water in China (Cr, Cu, and Pb ≤ 0.01 mg/L; Zn ≤ 0.05 mg/L; Ni is not included but the level ≤0.01 mg/L was selected as a criterion). The classes of WQI are uncontaminated, low, medium, and high for WQI values ≤ 1, 1 < WQI ≤ 2, 2 < WQI ≤ 3, and >3, respectively.

The geo-accumulation index (*Igeo*) has originally been used to assess the heavy metal pollution of sediments [17] and is defined as follows:

(2) $$I_{geo}=\log_{2}\frac{C_{j}}{kB_{j}},$$

where *C_j_* is the measured concentration of the sample, *B_j_* is the reference value, and *k* is the geo-accumulation constant (1.5) [18]; *B_j_* is identical with *C_R_*.

The *I_geo_* value of each heavy metal is classified into seven grades, ranging from uncontaminated to extremely contaminated (Table 1).

To further evaluate the degree of heavy metal contamination in sediments, the potential ecological risk index (RI), which was proposed by Hakanson [19], was developed based on the sedimentary theory. It can be used for the assessment of sediments and soil in large regional areas [20] and is calculated as follows:

(3) $$\mathrm{RI}=\sum_{i=1}^{n}T_{r}^{i}\times \frac{C_{j}^{i}}{C_{R}^{i}},$$

where *T*_r_ is the toxic-response factor and *C_R_* is the average background content of heavy metals for soil in Shandong Province, adopted for the present study [21].

Here, *T*_r_ accounts for the toxic requirement, and the sensitivity requirement is described as Cr (25) > Ni (10) > Cu = Pb (5) > Zn (1) after series statistic and standardization considering the pollution characteristics of four rivers in Linyi City [22,23].

The following terminology may be used to describe the risk factor:

RI < 150, low potential ecological risk;

150 ≤ RI < 300, moderate potential ecological risk;

300 ≤ RI < 600, high potential ecological risk;

RI > 600, very high ecological risk for the substance in question.

The bioconcentration factor (BCF) for metal uptake by plants was determined as a ratio of metal contents in each plant species. Because *P. crispus* is a rooted submerged angiosperm and *S. natans* is a free-floating aquatic plant, biological concentration factors were calculated on a dry weight (mg/kg) basis in different ways [24]:

BCF = Heavy metal_plant_/Heavy metal_sediment_ (for *P. crispus*)

BCF = Heavy metal_plant_/Heavy metal_water_ (for *S. natans*)

### 2.4. Statistical Analysis

The results presented are the arithmetic means with their corresponding standard deviations. Differences between groups were tested for significance via ANOVA, using the software package SPSS 17.0. According to Duncan’s multiple comparison tests, *p* < 0.05 was considered significant. Correlation coefficients of heavy metals between the plants and sediments (or water) were analyzed using the software package Excel.

## 3. Results

### 3.1. Distribution of Heavy Metals in Water and Sediment

As shown in Table 2, the concentrations of three metals (Cu, Zn, and Pb) in April met the standard of Class I (≤10 μg/L) for surface water in China, while the Cr level reached Class IV (10–50 μg/L); Ni is not included in the environmental quality standard for surface water in China. The mean metal concentration in the water samples decreased in the following order: Cr > Ni > Cu > Zn > Pb. Significant variations in the concentrations of metals were found among sites. The concentrations of Cr in the Beng River, the Su River, and the Yi River (D) were significantly higher (*p* < 0.05) than in the other two areas, while the Ni concentration was highest in the Liuqing River (*p* < 0.05). However, overall, no clear trend could be observed for the five areas.

Heavy metal concentrations, especially Cr, Ni, and Pb, were significantly lower (*p* < 0.05) in September than in April 2017 (Table 2). All the four heavy metals (Cr, Cu, Zn, and Pb) were in the range of Class I standard for surface water in China.

Table 3 shows the spatial distribution of the five heavy metals in the sediments of the four sections. The concentrations of all five heavy metals in the sediments were lower than the average background levels of heavy metals for the A horizon soil layer in Shandong Province, China. The Yi River (U) (section II) exhibited relatively low heavy metal levels, especially for Ni, Cu, and Pb. No significant differences were observed among the sediments from the sections I, III, and IV, which represented the Beng River, the junction of the four rivers, and the Yi River (D), respectively, except for significantly high concentrations of Cu and Pb in the sediment of the Yi River (D). The texture properties of the sediments are listed in Table 4. The results showed that the largest proportion (>80%) was sand in the samples from the four sections.

### 3.2. Risk Assessment of Heavy Metals in the Junction of the Four Rivers

#### 3.2.1. Water Quality Index

**Figure 2:**
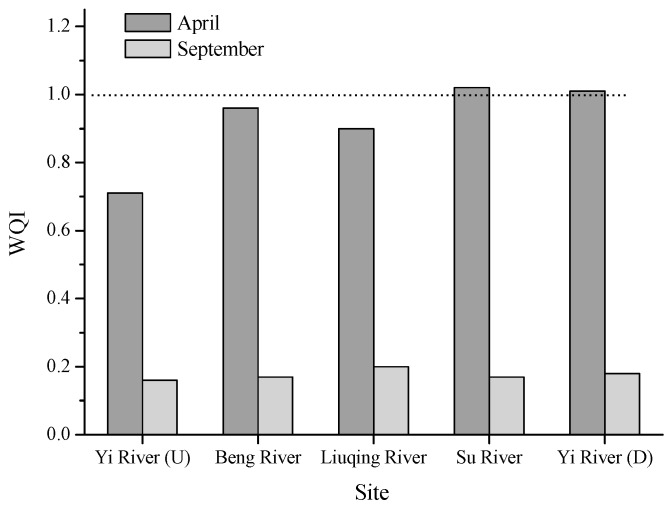
Water quality index (WQI) of heavy metals in the water samples in April and September, 2017.

The WQI serves as an index to determine the pollution levels of water bodies. Figure 2 shows the calculated WQI values in terms of heavy metals. In April, the values were significantly higher (three to four times) than in September. However, although the highest WQI values in April were found for the Su River (1.02) and for the Yi River (1.01), the pollution level was relatively low and followed the order Su River > Yi River (D) > Beng River > Liuqing River > Yi River (U). In September, the WQI levels were far below 1 for all areas, indicating negligible heavy metal pollution.

#### 3.2.2. Potential Ecological Risk Index (RI) and Geo-Accumulation Index (*I_geo_*)

The values of the *I_geo_* and the RI are summarized in Table 5; *I_geo_* was used to evaluate the pollution level in terms of single metals. Based on our results, the river sediments were largely uncontaminated (Class 0) as the *I_geo_* values of the metals were all below zero. The RI values were below 150, ranging from 12.21 to 16.84, which suggest a low ecological risk. In terms of the RI values, the four sections can be ranked in the following order: III > IV > II > I.

### 3.3. Heavy Metal Levels in Aquatic Macrophytes

Samples of the aquatic macrophyte *P. crispus*, which was only found in eight study sites, were collected to determine the concentrations of heavy metals. No distinct pattern was found (Table 6); however, the heavy metal concentrations in site 8, which were close to the junction of the four rivers, were significantly higher than those in the other sites (*p* < 0.05), except for Pb. Samples of *P. crispus* collected from site 1 of the Beng River accumulated large amounts of Ni and Pb. In contrast, for sites 2, 7, and 11, which were in the Beng River, the Yi River (U), and the Yi River (D), respectively, rather low heavy metal concentrations were detected. The heavy metal levels in the plant samples from site 12, in the Yi River (D), were moderate compared to those of the other sites.

Table 6 also shows the heavy metal concentrations of *S. natans*. The concentrations of Cr in site 1, Ni and Pb in site 11, and Zn in site 2 were significantly higher (*p* < 0.05) than those in the other sites (26.88 mg/kg Cr, 5.86 mg/kg Ni, 6.57 mg/kg Pb, 46.45 mg/kg Zn). No significant differences (*p* < 0.05) in the concentrations of Cu were found among all sites.

### 3.4. Bioconcentration of Heavy Metals by Aquatic Plants

The BCF of heavy metals in *P. crispus*, which was calculated based on the metal levels in the plants and sediment, is presented in Table S3. Overall, the BCF values of heavy metals in *P. crispus* did not vary considerably among the eight sites in the Beng River, Yi River (U), and Yi River (D). The values of BCF of all five metals in the plants from sites 2, 7, and 11 were lower than those from plants of the other five sites. Compared to the other heavy metals, the plants showed a relatively poor bioconcentration capacity for Pb. For *S. natans*, the bioaccumulation capacity was relatively high given the low metal concentrations of the water, with the highest accumulation for Pb (190,200) at site 11 (Table S4). The BCF values followed the order Ni < Cr < Cu < Zn < Pb, with average values of 2.3, 4.3, 12.3, 29.0, and 73.2, respectively. Similar to *P. crispus*, BCF of heavy metals in *S. natans* did not show any spatial differences.

**Figure 3:**
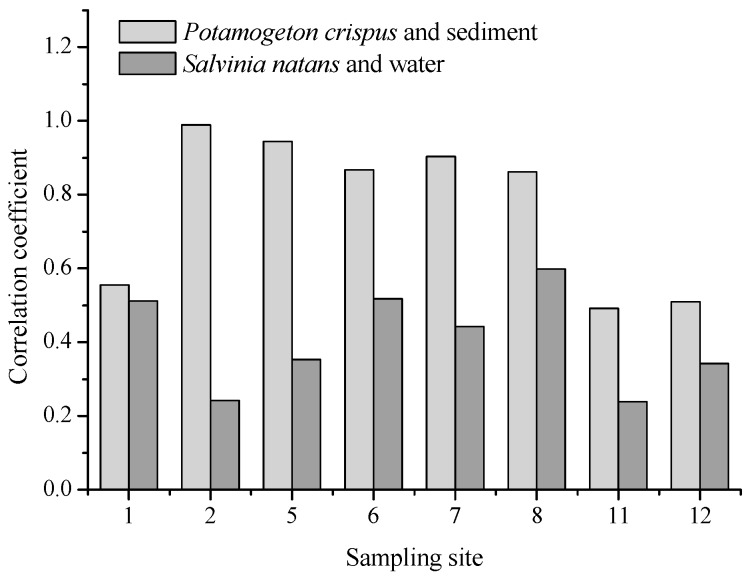
Correlation coefficients of heavy metals between the plants and water (or sediments).

The correlation coefficients of the five heavy metals between *P. crispus* and the sediments of the corresponding sampling sites (Table S5) are listed in Figure 3. Five of the eight study sites showed high correlation coefficients (beyond 0.8). However, the correlation coefficients of the metals between *S. natans* and water of the corresponding sampling sites were below 0.6 for all sites.

## 4. Discussion

As a seasonal river in Northern China, the Yi River has different flow rates in different seasons. More than 80% of the annual distribution of runoff are concentrated in the flood season (June to September) [25]. The river runs through Linyi City, and three tributaries feed into in the northeastern part of the City. These two factors inevitably influence the distribution of pollutants, including heavy metals, in the junction of the four rivers. In addition, the rubber dam built downstream in 1997 has created a lake near the junction of the four rivers, which strongly influenced the distribution and deposition of the pollutants released into the upstream.

In the present study, we selected the months of April and September for sample collection, mainly because the river experienced a long-time dry season in April, which probably resulted in the concentration of pollutants, and a three-month flood season in September, with a subsequent dilution. Our results clearly show that the concentrations of the four metals (Cr, Ni, Zn, and Pb) decreased significantly (*p* < 0.05) in September compared to April (Table 2). Consequently, the calculated WQI values decreased from about 1 to far below 1 (Figure 2), indicating an improved water quality. During the dry season of the rivers, Cr was the main factor contributing to the low water quality and high toxicity, and therefore, the bioaccumulation in aquatic organisms deserves more attention. The element Cr may come from the chemical and electroplating factories. In addition, petroleum chemical industry, agriculture production and the agricultural sideline products can result in Cr accumulation in rivers [26]. In both months, the Cu levels did not significantly fluctuate.

The pollution of sediments with heavy metals can seriously degrade aquatic ecosystems, and their concentrations are considered as reliable indicators of ecosystem health [27]. The results of the present study show that the concentrations of Cr, Cu, Zn, and Pb in the sediments were rather low compared with several rivers in China, such as the Yangze River, the Huaihe River, the Luan River, and the Second Songhua River, with reported maximum concentrations of 205.00, 178.61, 1142.00, and 113.00 mg/kg for Cr, Cu, Zn, and Pb, respectively (Table 7). The concentrations of Cu, Zn, and Pb in the Yi River were also lower than those of Gomti River, Po River, Almendares River, and Lahn River. Compared with the guidelines established by the New York State Department of Environmental Conservation [28], which proposed the lowest effect screening levels for Cr, Ni, Cu, Zn, and Pb of 26, 31, 16, 120, and 16 mg/kg, respectively, the heavy metal levels in all sites of this study did not exceed the upper threshold values, except for Cu and Pb in the Yi River (D) (section IV, Table 3), which probably originated from industries, atmospheric deposition, and river borne sources [29,30]. Accordingly, the results of the potential ecological risk analysis showed that the sediments in the four sections were uncontaminated, with a low pollution risk, which was reflected by the *I_geo_* and RI values, respectively. The ability of sediments to accumulate heavy metals is affected by the sediment composition [31,32]. The sediment samples collected in this study contained more than 80% of sand, which might explain the low metal concentrations. In addition, the Yi River is an important flood discharge river. The annual runoff in the Yi River was 1.124 billion m^3^ in 2017, with 60% of which concentrated in July and August [33]. The scouring function of flood may also reduce the deposition of pollutants in the sediments.

The two aquatic macrophytes studied, *P. crispus* and *S. natans*, are widely distributed depending on the season, with high growth rate in the Yi River and the Beng River. Investigations on the bioaccumulation potential of these plants may be significant for biomonitoring studies and can provide reference data for the further development of phytoremediation technologies. The two plant species take up nutrients and pollutants differently. While *P. crispus* is a rooted submerged angiosperm which extracts nutrients and heavy metals mostly from sediments via root hairs and from the surroundings [41], *S. natans* absorbs nutrients from the surface water as a free-floating aquatic plant. Therefore, the BCF values of *P. crispus* and *S. natans* were calculated in this study based on the metal concentrations in the surrounding sediment and water, respectively.

Aquatic macrophytes, including *P. crispus* and *S. natans*, are considered resistant to heavy metals and may accumulate large amounts of heavy metals in their tissues [42,43]. Therefore, these species are highly suitable for the use as bio-indicator organisms of heavy metal pollution. While *P. crispus* has shown potential to accumulate considerable amounts of Cu, Pb, Ni, and Zn [44], *S. natans* is a hyperaccumulator for Cr, Ni, Cu, and Zn, accumulating amounts of up to 0.5% of its dry weight [45]. This plant species can remove more than one metal when exposed to multi-metal solutions [46]. In the present study, both species showed an excellent ability to accumulate heavy metals (Table 6, Tables S3 and S4). Because *P. crispus* grew in a fixed region with its roots in the sediment and lived through a long-term dry season, it can also be used as a bio-indicator reflecting the heavy metal pollution of sediments. We found a high correlation between the heavy metal concentrations of *P. crispus* and the sediments of the corresponding sampling sites (Table 5). In September 2017, *P. crispus* could not be collected in most of the sampling sites because of increasing water levels, while *S. natans* could travel a long distance, especially in the flood season, and its growth period in a specific area could therefore not be determined. In addition, this plant appeared in the Yi River and the Beng River, with relatively high heavy metal levels before the flood season. Hence, the potential of *S. natans* as a bio-indicator is relatively low. The BCF values of heavy metals in *S. natans* were calculated based on the low metal concentrations of water in September (compared with April), and thus, the bioaccumulation capability of *S. natans* may be overrated. The factors that influence metal accumulation are plant species, environmental conditions, and the surrounding metal levels. Both environmental conditions and the metal level in water or sediments of the three areas (Beng River, Yi River (U), and Yi River (D)) did not vary greatly. This may be the main reason for no spatial differences of the BCF values. Kastratović et al. observed that BCF of Ni and Sr in *Lemna minor* from Lake Skadar in Montenegro had no spatial differences [47]. The two plants selectively accumulated heavy metals; while *P. crispus* accumulated higher levels of Cr, Ni, Cu, and Zn, *S. natans* showed a high accumulation efficiency for Pb and Zn. Although aquatic plants show high accumulation capacity for specific heavy metals, the metals would release from the plant tissues when the plants die at the end of the growing season, and then return into the water body or sediment. This process should be paid enough attention in phytoremediation using aquatic plants.

## 5. Conclusions

The concentrations of the heavy metals Cu, Zn, and Pb in water samples were below the first-grade levels outlined in the China Environmental Quality Standards for Surface Water, suggesting a low pollution. The sediment in the junction of the rivers was uncontaminated (Class 0) on the basis of *I_geo_* and RI values. Spatial distribution showed that heavy metal concentration of water and sediment in different areas remained almost constant, except for the relatively low level in the Yi River (U), strongly suggesting that the inflow of the tributaries increased the risk of heavy metal concentration. The aquatic plant species *P. crispus* accumulated considerable amounts of heavy metals, and there was a correlation between plant and sediment metal concentrations. This indicates that *P. crispus* is a suitable bio-indicator for heavy metal contamination of sediments. Our work offers information for the Yi River basin management as well as for phytoremediation using aquatic plants.

## Figures and Tables

**Table 1:** Classification of heavy metal pollution in sediment based on *I_geo_* value.

Risk Level	Range of *I_geo_*	Pollution Degree
0	*I_geo_* ≤ 0	uncontaminated
1	0 < *I_geo_* ≤ 1	uncontaminated to moderately contaminated
2	1 < *I_geo_* ≤ 2	moderately contaminated
3	2 < *I_geo_* ≤ 3	moderately to heavily contaminated
4	3 < *I_geo_* ≤ 4	heavily contaminated
5	4 < *I_geo_* ≤ 5	heavily to extremely contaminated
6	5 < *I_geo_* ≤ 10	extremely contaminated

**Table 2:** Heavy metal levels of the water samples in April and September, 2017. (μg/L).

River	Month	Cr	Ni	Cu	Zn	Pb
Yi River (U)	April	28.29 ± 2.70 ^b^	3.99 ± 1.07 ^b^	2.18 ± 0.68 ^b^	0.76 ± 0.44 ^c^	0.71 ± 0.20 ^b^
	September	5.05 ± 0.55 ^b,^*	1.32 ± 0.23 ^b,^*	1.18 ± 0.34 ^b^	1.24 ± 0.37 ^b,c^	0.10 ± 0.04 ^a,b,^*
Beng River	April	39.57 ± 7.05 ^a^	5.55 ± 3.29 ^b^	1.72 ± 0.55 ^b^	1.24 ± 0.70 ^b,c^	0.77 ± 0.27 ^a^
	September	5.87 ± 0.41 ^a,^*	1.32 ± 0.29 ^b,^*	1.17 ± 0.20 ^b^	1.39 ± 0.12 ^a,b^	0.05 ± 0.01 ^c,^*
Liuqing River	April	25.84 ± 3.99 ^b^	14.65 ± 2.74 ^a^	3.40 ± 1.19 ^a^	1.69 ± 1.32 ^a,b^	0.70 ± 0.11 ^b^
	September	4.99 ± 1.36 ^b,^*	2.57 ± 1.68 ^a,^*	1.92 ± 1.15 ^a^	1.62 ± 0.35 ^a^	0.14 ± 0.08 ^a,^*
Su River	April	42.88 ± 10.40 ^a^	4.31 ± 2.21 ^b^	2.48 ± 1.28 ^a,b^	2.34 ± 0.50 ^a^	1.02 ± 0.33 ^a^
	September	6.07 ± 0.23 ^a,^*	1.36 ± 0.43 ^b,^*	1.04 ± 0.36 ^b^	1.47 ± 0.17 ^a,b,^*	0.06 ± 0.01 ^bc,^*
Yi River (D)	April	41.25 ± 8.18 ^a^	6.15 ± 2.45 ^b^	2.09 ± 0.45 ^b^	0.82 ± 0.25 ^c^	1.05 ± 0.20 ^a^
	September	6.06 ± 0.33 ^a,^*	1.64 ± 0.14 ^b,^*	0.91 ± 0.10 ^b,^*	1.05 ± 0.21 ^c^	0.04 ± 0.01 ^c,^*

Values represent mean and standard deviation. In each column, different superscript letters indicate significant difference (*p* < 0.05) between sampling sites for the same heavy metal and same sampling time; “*” means significant difference (*p* < 0.05) between different months for the same metal and sampling site.

**Table 3:** Heavy metal levels of sediment from four sections and the average metal levels in the A horizon soil layer in Shandong Province, China. (mg/kg).

Section	Cr	Ni	Cu	Zn	Pb
I	15.79 ± 1.41 ^a^	12.08 ± 1.97 ^a^	13.43 ± 3.81 ^a,b^	24.27 ± 1.59 ^a^	9.92 ± 1.76 ^b^
II	12.51 ± 3.00 ^a^	7.60 ± 0.83 ^b^	8.72 ± 0.63 ^b^	19.79 ± 6.47 ^a^	8.65 ± 2.41 ^b^
III	14.10 ± 1.38 ^a^	13.75 ± 1.61 ^a^	10.06 ± 3.32 ^b^	20.97 ± 2.26 ^a^	8.50 ± 0.83 ^b^
IV	14.08 ± 2.37 ^a^	6.53 ± 0.95 ^b^	16.77 ± 1.07 ^a^	21.89 ± 1.70 ^a^	17.93 ± 3.45 ^a^
A	66.0 ± 14.8	25.8 ± 9.0	24.0 ± 9.8	63.5 ± 18.2	25.8 ± 8.6

In each column, the data with the same superscript letter indicate no significant difference (*p* > 0.05). “A” means the A horizon soil layer in Shandong Province, China.

**Table 4:** The textural properties of the sediment samples.

Section	Sand (%)	Silt (%)	Clay (%)
I	81.1	10.5	8.4
II	83.5	14.2	2.3
III	84.3	8.6	7.1
IV	80.6	12.3	7.1

**Table 5:** The geo-accumulation index (*I*_geo_) and risk index (RI) values for the sediments of the junction of the four rivers in Linyi City.

Section	*I*_geo_					RI
	Cr	Ni	Cu	Zn	Pb	
1	−2.63	−1.51	−1.28	−1.94	−1.88	12.21
2	−2.97	−2.18	−1.90	−2.23	−2.08	15.79
3	−2.79	−1.33	−1.69	−2.15	−2.10	16.84
4	−2.80	−2.40	−0.96	−2.08	−1.02	16.14

**Table 6:** Heavy metal levels of *Potamogeton crispus* and *Salvinia natans*. (mg/kg).

Species	Site	Cr	Ni	Cu	Zn	Pb
*P.* *crispus*	1	99.86 ± 1.65 ^b^	142.65 ± 7.63 ^b^	97.42 ± 2.18 ^c^	359.86 ± 5.94 ^c^	92.11 ± 0.76 ^a^
	2	89.42 ± 2.82 ^b^	22.74 ± 5.21 ^e^	66.87 ± 8.26 ^d^	445.71 ± 77.02 ^b^	18.35 ± 1.86 ^c^
	5	108.10 ± 9.10 ^b^	61.18 ± 23.99 ^d^	122.17 ± 4.64 ^b^	282.74 ± 107.37 ^c^	32.36 ± 10.42 ^b,c^
	6	110.38 ± 37.74 ^b^	111.52 ± 0.70 ^c^	123.48 ± 2.57 ^b^	313.21 ± 23.64 ^c^	17.82 ± 1.66 ^c,d^
	7	87.10 ± 8.25 ^b^	54.79 ± 19.86 ^d^	75.86 ± 2.28 ^d^	400.05 ± 53.35 ^b^	11.35 ± 2.08 ^d^
	8	350.10 ± 48.72 ^a^	242.66 ± 18.37 ^a^	226.76 ± 32.87 ^a^	736.79 ± 38.56 ^a^	35.08 ± 5.51 ^b^
	11	84.90 ± 16.59 ^b^	94.78 ± 9.71 ^c^	69.20 ± 5.47 ^d^	304.69 ± 9.39 ^c^	18.37 ± 11.04 ^c^
	12	133.98 ± 5.92 ^b^	113.03 ± 10.77 ^c^	104.26 ± 8.31 ^c^	353.42 ± 86.00 ^c^	39.75 ± 6.01 ^b^
*S. natans*	1	26.88 ± 1.50 ^a^	3.29 ± 0.04 ^b,c^	12.00 ± 0.19 ^a^	34.47 ± 1.10 ^b,c^	4.27 ± 0.15 ^b^
	2	21.16 ± 0.50 ^c,d^	2.99 ± 0.13 ^c^	10.15 ± 0.38 ^a^	46.45 ± 1.71 ^a^	4.78 ± 0.60 ^b^
	5	24.97 ± 2.00 ^a,b^	3.58 ± 0.31 ^b^	24.24 ± 9.75 ^a^	37.12 ± 1.99 ^b^	3.72 ± 0.30 ^c,d^
	6	23.09 ± 0.62 ^b,c^	2.88 ± 0.42 ^c^	12.61 ± 0.92 ^a^	31.45 ± 2.73 ^c^	3.19 ± 0.02 ^d^
	7	23.38 ± 0.15 ^b,c^	3.13 ± 0.05 ^b,c^	12.04 ± 0.89 ^a^	30.54 ± 1.60 ^c^	3.46 ± 0.41 ^d^
	8	20.19 ± 0.64 ^d^	1.82 ± 0.34 ^e^	8.84 ± 1.24 ^a^	20.06 ± 0.64 ^d^	2.00 ± 0.17 ^e^
	11	23.5 ± 0.96 ^b,c^	5.86 ± 0.21 ^a^	15.24 ± 0.78 ^a^	40.86 ± 0.73 ^b^	6.57 ± 0.44 ^a^
	12	21.80 ± 0.09 ^c,d^	2.69 ± 0.03 ^d^	11.20 ± 0.80 ^a^	31.91 ± 0.84 ^c^	3.40 ± 0.15 ^d^

**Table 7:** Maximum Cr, Ni, Cu, Zn, and Pb concentrations in sediments of the selected water bodies in China from literature.

Site	Maximum Concentration (mg/kg)					Reference
	Cr	Ni	Cu	Zn	Pb	
Yi River	15.79	13.75	16.77	24.27	17.93	This study
Yangtze River, China	205.00	-	129.00	1142.00	98.00	[34]
Huaihe River, China	73.70	-	54.60	83.10	113.00	[35]
Luan River, China	152.73	-	178.61	22.56	38.29	[36]
Second Songhua River, China	69.6	27.6	75.4	150.5	37.0	[37]
Gomti River, India	19.13	-	35.03	101.70	75.30	[27]
Po River, Italy	-	-	90.10	305.00	98.50	[38]
Almendares River, Cuba	23.40	-	420.80	708.80	189.00	[39]
Lahn River, Germany	-	-	48.20	245.20	68.40	[40]

## Supplementary Materials

The following are available online at https://www.mdpi.com/1660-4601/16/16/2861/s1, Table S1: Longitude and latitude of the water sampling sites; Table S2: Longitude and latitude of the sediment sampling sites; Table S3: Bioconcentration factors of *Potamogeton crispus* in April, 2017; Table S4: Bioconcentration factors (×10^3^) of *Salvinia natans* in September, 2017; Table S5: Heavy metal levels in sediment of eight sites in April, 2017 (mg/kg).
