# Supplementary material for: Distribution and Potential Ecological Risk of Heavy Metals in Water, Sediments, and Aquatic Macrophytes: A Case Study of the Junction of Four Rivers in Linyi City, China
Source: Int J Environ Res Public Health. 2019 Aug 10;16(16):2861. doi: 10.3390/ijerph16162861 (PMC6719217; doi:10.3390/ijerph16162861)
Supplement: Supplementary file 1 [file ijerph-16-02861-s001.pdf]

Supplementary material

Distribution and potential ecological risk of heavy metals in water, sediments, and aquatic macrophytes: A case study of the junction of four rivers in Linyi City, China

July 9, 2019

Submitted to: *International Journal of Environmental Research and Public Health*

Three pages

Five Tables

Contents

S1 Longitude and latitude of the water sampling sites.

S2 Longitude and latitude of the sediment sampling sites.

S3 Bioconcentration factors of *Potamogeton crispus* in April, 2017.

S4 Bioconcentration factors (× 10^3) of *Salvinia natans* in September, 2017.

S5 Heavy metal levels in sediment of eight sites in April, 2017. (mg/kg)

Table S1. Longitude and latitude of the water sampling sites.

Sampling sites	Longitude	Latitude
1	118º 21' 27.38''	35º 04' 5.71''
2	118º 20' 48.24''	35º 05' 28.82''
3	118º 21' 50.61''	35º 04' 59.23''
4	118º 22' 01.49''	35º 05' 21.41''
5	118º 22' 24.56''	35º 05' 06.11''
6	118º 22' 43.49''	35º 06' 18.96''
7	118º 22' 48.02''	35º 04' 59.51''
8	118º 22' 33.82''	35º 04' 13.56''
9	118º 21' 31.80''	35º 04' 24.67''
10	118º 20' 56.07''	35º 04' 36.92''
11	118º 21' 44.90''	35º 04' 04.06''
12	118º 22' 55.10''	35º 03' 07.52''

Table S2. Longitude and latitude of the sediment sampling sites.

Sampling sites		Longitude	Latitude
	1	118º 22' 16.82''	35º 04' 23.37''
Section I	2	118º 22' 12.04''	35º 04' 19.25''
	3	118º 21' 18.92''	35º 04' 12.99''
	1	118º 21' 20.95''	35º 04' 50.48''
Section II	2	118º 21' 26.33''	35º 04' 54.52''
	3	118º 21' 29.66''	35º 04' 56.60''
	1	118º 22' 31.46''	35º 05' 08.28''
Section III	2	118º 22' 48.35''	35º 05' 08.83''
	3	118º 22' 51.55''	35º 05' 09.73''
	1	118º 22' 21.65''	35º 02' 35.30''
Section IV	2	118º 22' 34.75''	35º 02' 38.44''
	3	118º 22' 54.61''	35º 02' 46.98''

Table S3. Bioconcentration factors of *Potamogeton crispus* in April, 2017.

Site	Cr	Ni	Cu	Zn	Pb
1	9.9	14.9	10.3	19.3	4.0
2	6.0	1.8	4.7	17.4	1.7
5	11.6	10.2	13.7	14.1	3.8
6	10.4	15.7	12.5	19.8	2.0
7	6.1	6.8	9.3	19.9	1.3
8	13.9	14.7	13.9	28.8	3.9
11	5.5	12.2	4.1	13.5	1.0
12	9.7	17.9	6.9	17.3	2.4
average	9.2	11.8	9.4	18.8	2.7

Table S4. Bioconcentration factors (× 10^3) of *Salvinia natans* in September, 2017.

Site	Cr	Ni	Cu	Zn	Pb
1	4.9	2.3	9.8	25.4	100.3
2	3.4	2.5	9.1	32.5	94.3
5	5.8	2.6	21.8	28.3	25.2
6	4.7	2.4	8.0	21.7	26.4
7	4.5	2.6	10.4	33.9	45.5
8	3.6	1.2	10.4	20.7	32.2
11	3.7	3.4	16.5	33.9	190.2
12	3.8	1.8	12.5	35.8	71.6
average	4.3	2.3	12.3	29.0	73.2

Table S5. Heavy metal levels in sediment of eight sites in April, 2017. (mg/kg)

Site	Cr	Ni	Cu	Zn	Pb
1	10.11±0.59	9.65±0.85	9.59±2.35	18.62±1.35	18.33±1.45
2	14.93±1.02	12.35±1.22	14.25±0.76	25.67±2.93	10.54±1.52
5	9.35±1.21	6.04±0.86	8.93±1.05	20.19±3.12	8.58±0.86
6	10.66±0.67	7.11±0.77	9.92±0.77	15.87±2.18	8.88±1.66
7	14.35±2.14	8.00±1.53	8.25±1.31	20.18±1.11	8.66±1.72
8	25.13±3.01	16.54±1.28	16.34±3.07	25.68±3.56	9.18±2.05
11	15.33±0.87	7.84±0.74	16.96±2.21	22.51±2.45	18.20±1.11
12	13.87±1.45	6.33±0.68	15.25±1.69	20.41±2.77	16.52±3.58
